# NTPDase5/PCPH as a New Target in Highly Aggressive Tumors: A Systematic Review

**DOI:** 10.1155/2014/123010

**Published:** 2014-06-23

**Authors:** Paula Andreghetto Bracco, Ana Paula Santin Bertoni, Márcia Rosângela Wink

**Affiliations:** Laboratório de Biologia Celular, Departamento de Ciências Básicas da Saúde, Universidade Federal de Ciências da Saúde de Porto Alegre (UFCSPA), Rua Sarmento Leite, Rua Sarmento Leite, No. 245, 90050-170 Porto Alegre, RS, Brazil

## Abstract

The protooncogene *PCPH* was recently identified as being the ectonucleoside triphosphate diphosphohydrolase 5 (*ENTPD5*). This protooncogene is converted into an oncogene by a single base pair deletion, resulting in frame change and producing a premature stop codon, leading to a mutated protein (mt-PCPH) with only 27 kDa, which is much smaller than the original 47 kDa protein. Overexpression of the PCPH as well as the mutated PCPH increases the cellular resistance to stress and apoptosis. This is intriguing considering that the active form, that is, the oncogene, is the mutated PCPH. Several studies analyzed the expression of NTPDase5/mt-PCPH in a wide range of tumor cells and evaluated its role and mechanisms in cancer and other pathogenic processes. The main point of this review is to integrate the findings and proposed theories about the role played by NTPDase5/mt-PCPH in cancer progression, considering that these proteins have been suggested as potential early diagnostic tools and therapy targets.

## 1. Introduction

The ectonucleoside triphosphate diphosphohydrolase 5 (NTPDase5; EC: 3.6.1.6), also known as CD39L4 (*CD39 antigen-like 4*) [1], is an enzyme that acts mostly on diphosphate nucleosides, rather than triphosphate nucleosides. It can be secreted from mammalian cells through an aminoterminus hydrophobic domain that encodes a signal peptide sequence [2]. The CD39L4 has three potential glycosylation sites and has the capacity to form disulfide dimers; however, none of these characteristics seem to significantly contribute to the ADPase or UDPase activity, which distinguishes the CD39L4 from the other proteins in this family [3]. The NTPDase5 also differs from the other members of E-NTPDase family, as it is the only one characterized as a protooncogene, also known as PCPH [4, 5].

The* PCPH* is a highly conserved gene from yeast to humans, suggesting basic roles in eukaryotic cells function [6]. It is located on chromosomes 12 and 14 in mice and humans, respectively, and it is transformed into an oncogene, called* mt-PCPH,* through a single base pair depletion resulting in frame change and producing a premature stop codon, originating a protein with 246 amino acids (25 kDa) instead of the normal protein with 469 amino acids (47 kDa) [7].

In the first studies, it was not possible to conclude whether the PCPH had nucleotide degrading activity, since it shares the apyrase conserved regions [7]. In 2001, Páez and collaborators showed a degree of genetic similarity of 98% between the protooncogene* PCPH* and* ENTPD5* and also determined that PCPH and NTPDase5 were capable of degrading ADP and ATP (preferentially ADP) [4]. First, it was shown that extracts from stable mt-PCPH-transformed NIH3T3 cells presented GDPase activity, using in-gel activity (performed by native gel electrophoresis of the extracts and staining for inorganic phosphate) and standard GDPase assays [8]. Later, the same group, by a different experimental approach, demonstrated that NTPDase activity of mt-PCPH was undetectable* in vitro* or when tested* in situ* in living cells [9]. The authors stated that this discrepancy could be due to the production of recombinant mt-PCPH in non-human expression systems.

The* PCPH* gene was initially identified as a protooncogene, due to its frequent mutation in the 3-methylcholantrene-induced tumorigenesis process in a Syrian hamster fibroblast cell line [6]. Subsequent studies were mainly based on the hypothesis that the carcinogenic action of PCPH/NTPDase5 could modulate the cellular balance of ATP, potentially interfering with cellular functions such as cell cycle, apoptosis, autophagy, proliferation, and differentiation [5].

The major functional difference between the normal NTPDase5 and the mt-PCPH oncoprotein is that the former provides lower levels of protection against apoptotic agents, including chemotherapeutic drugs, and radiation than the latter. This role of mt-PCPH seems to be mediated by its ability to promote a Ras-independent sustained activation of the ERK pathway [7, 8, 10]. The different expression profiles of these proteins and consequent changes in the cellular resistance observed in the studies reviewed suggest that it may be involved in the survival, growth, or migratory activity of some kinds of tumors. Yet, it is not very clear how this protein, in fact, contributes to the development of the different types of neoplasias. In addition, it was observed that NTPDase5 KO mice presented an increase in cases of liver pathology and neoplasia [11], adding more doubts about how the level of its expression influences cancer development.

The aim of this work is to provide an overview, in a systematic review format, of the expression profile of the NTPDase5 and mt-PCPH in cancer cell lines and tumor samples, in comparison to healthy tissues, and describe the proposed mechanisms by which the mutated and WT proteins act in the neoplastic development.

## 2. Method

This review describes a “literature overview” about PCPH/NTPDase5 in all types of cancer.

### 2.1. Search Details

**Figure 1:**
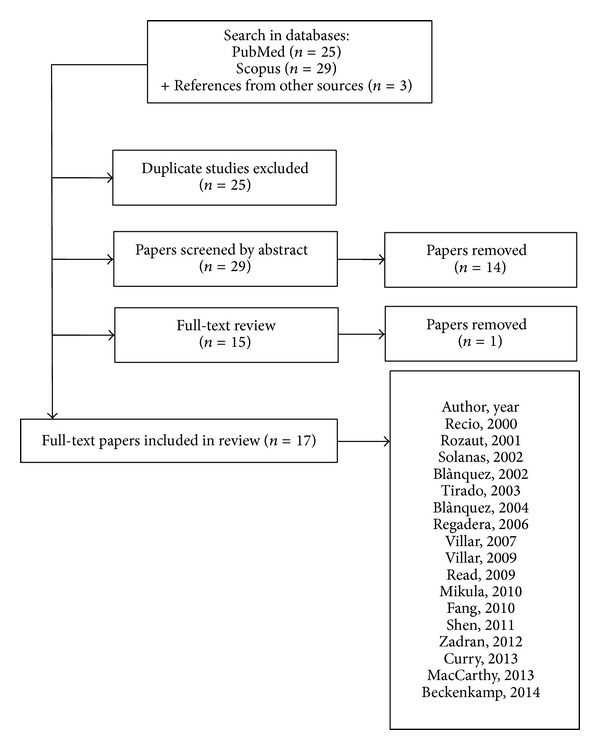
Methodological flow chart of the search strategy in PubMed and Scopus databases.

We performed an electronic search on January, 2014, for papers indexed in PubMed and Scopus database. The search strategy comprised only the medical subject heading (MeSH) term “ENTPD5 or NTPDase5.” For inclusion in this review, papers had to describe any relation of PCPH/NTPDase5 to cancer. No language restriction was applied. By this search strategy, 55 papers were identified. After reviewing their abstracts, 14 eligible papers were chosen and three citations retrieved from manual search were included, providing 17 papers that examined PCPH/NTPDase5 expression status in cancer (Figure 1).

Data were extracted from each original study about PCPH/NTPDase5 gene or protein expression in normal and pathological state as well as its isoform expression patterns in tissues and tumor cells.

## 3. Results and Discussion

### 3.1. Expression Profile of ENTPDase5

*ENTPD5* is expressed at different levels in several types of normal and neoplastic tissues suggesting a tissue-specific regulation [12, 13]. Interestingly, the first studies on this subject revealed the presence of 27 kDa PCPH immune-related polypeptide (believed to be the truncated, i.e., mutated,* PCPH* oncogene) in various cell extracts of healthy epithelial tissues and the expression of 47 kDa polypeptide (believed to be the full length* PCPH* gene), in most of the tumor cell lines studied, hypothesizing that there was a potential relationship between the levels of these proteins and the neoplastic phenotype [12]. However, high levels of the normal NTPDase5 have also been identified in healthy liver and kidney tissues [13]. It is important to mention that these studies were performed with an anti-PCPH polyclonal antiserum raised in rabbits with a purified bacterial recombinant PCPH [8, 12, 13]. Considering that the PCPH/NTPDase5 shares the conserved apyrase regions [1], it is possible that this antiserum also recognizes other members of the E-NTPDase family.

Thus, it is possible to conclude that the expression of ENTPD5/mt-PCPH in tumors studied so far is highly variable, as described in Tables 1 and 2.

Breast tumors induced in mice exhibited increased expression of the normal NTPDase5 and decreased expression of the mt-PCPH when compared with healthy mammary gland, and this difference was more evident in malignant tumors in comparison to benign tumors [13]. A similar expression profile was observed when comparing neoplastic cell lines of the larynx and healthy cells from a primary culture of the larynx. A higher mt-PCPH expression was observed in the healthy cells when compared with carcinoma cells lines: UM-SSC-11, UM-SCC-12, UM-SCC-23, and SCC-81B. Interestingly, a loss of this protein expression was associated with an increase in the expression of the normal NTPDase5 [19]. In human normal and neoplastic breast samples, however, the normal NTPDase5 appeared in all tested samples both benign and malignant. Expression of mt-PCPH correlated positively with the aggressiveness of the breast carcinoma and was not detected in the benign tumors [18].

Testicular germ tumor cell lines NCCIT and NT2/D1 also presented high levels of NTPDase5 and very low levels of mt-PCPH [17]. However, most clinical specimens of germ tumor cell of 54 patients showed an increase in expression of mt-PCPH when compared with healthy adjacent tissue [17]. Additionally, the expression of mt-PCPH is increased in the precursor lesions of germ tumor cell.

Immunohistochemical analysis also identified that, in cases of laryngeal cancer and testicular germ tumor cells, the concentration of NTPDase5 is higher in areas of differentiation and neoplastic transformation, with low proliferation, than in areas with high proliferation rates, raising the hypothesis that this protein acts more in the initial processes of cancer than in well-advanced, malignant neoplastic phenotypes [17, 19].

Studies with clinical specimens of prostate cancer and prostate tumor cell lines RWPE-1, LNCaP, C4-2, and PC-3 demonstrated that NTPDase5 is not significantly expressed in healthy prostate tissue but is present in cases of benign hyperplasia and is more pronouncedly expressed in tumor samples. Also, this protein was not detected in the tumor cell line PC-3 but was expressed in LNCaP cell line. In addition, a positive relationship between the level of NTPDase5 expression, and especially mt-PCPH expression, and the invasiveness of prostate cancer was found, thus associating the expression of the NTPDase5 more with cancer motility than with its proliferation [16].

Clinical samples of glioblastoma multiforme (GBMs) showed a higher level of NTPDase5 expression when compared to adjacent normal tissues [15]. In addition, the samples with higher expression levels of this protein were associated with significantly lower survival rates when compared to samples with lower NTPDase5 expression. However, the* NTPDase5* expression profile in individual samples of normal colon, adenomas, and colon adenocarcinomas presented that the* ENTPD5* gene expression decreased with the increase of lesion malignancy [20].

When an increase in either the NTPDase5 or the mt-PCPH expression was present, this was observed as early as nonneoplastic lesions, suggesting that the deregulation of these proteins is involved in the initial stages of neoplastic development. Thus, it is possible to consider the use of the NTPDase5 as a tool for early identification of various neoplastic cells [13, 16, 17].

Recently, cervical human cancer cells SiHa (HPV 16-positive), HeLa (HPV 18-positive), and C33A (HPV-negative) were shown to present different levels of* ENTPD5* gene expression, and the highest expression observed in SiHa cells suggests a link between* ENTPD5* and oncogenic viral proteins in cervical cancer development [14].

### 3.2. How NTPDase5 Acts in Cancer Progression

The neoplastic transforming activity is the role of the mt-PCPH that represents the major functional difference between the normal protooncogene and mutated active oncogene. It is supposed that this activity is due to the ability of this protein to cause a Ras-independent sustained activation of ERK1 [8].

Although the normal NTPDase5 lacks the transforming ability, it is suggested that the NTPDase5 and especially the mt-PCPH confer resistance to cells subjected to stress conditions [7]. Such resistance is conferred in part by the diphosphohydrolase activity of this enzyme, which causes a reduction in the intracellular ATP and inactivation of the stress-activated protein kinases, which is reversed by returning the intracellular ATP to physiological levels [21].

**Figure 2:**
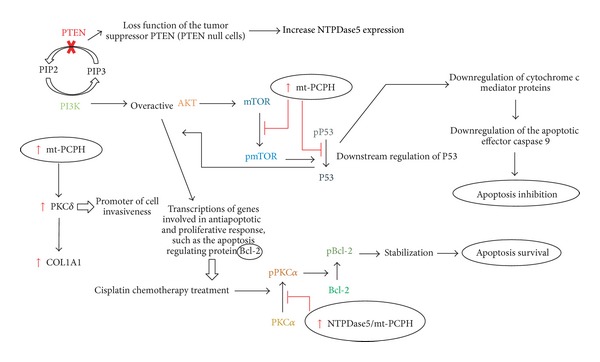
Integration of the proposed pathways by which the NTPDase5/mt-PCPH acts on the neoplastic progression. Due to the lack of information about the role of these proteins in cancer development and progression, this scheme presents all data published so far, not taking into consideration in which cell the proposed mechanisms were studied although it is possible that some of the contradictions presented may be a direct consequence of this fact. The figure demonstrates how the loss of the tumor suppressor PTEN possibly causes an increase in NTPDase5 expression and an overactive PI3K/AKT pathway. AKT and mTOR regulate cell growth and survival, such as Bcl-2 gene leading to an increase in apoptotic resistance. Furthermore it is also suggested that the NTPDase5 interacts with PKC*δ*, upregulating its levels and inducing cancer cell invasiveness. pP53, pPKC*α*, and pBcl-2 are the phosphorylated forms of the respective proteins and correspond to the phosphorylation of p53 at Ser^18^, pPKC at Trh^638^, and Bcl-2 at Ser^70^.

One of the cell protection mechanisms against apoptosis afforded by NTPDase5 is through inhibition of mTOR. After cellular exposure to ionizing radiation, mTOR plays a proapoptotic role and this role is antagonized by the expression of mt-PCPH protein or by the overexpression of the normal protein NTPDase5. They are responsible for blocking the activation of mTOR and its translocation from the cytoplasm to the nucleus, preventing the phosphorylation of p53 at Ser^18^. Phosphorylation of p53 mediates the release of cytochrome c by mitochondria and the subsequent activation of caspase 9/3, inducing the apoptosis [8]. This signaling pathway is influenced by intracellular ATP concentrations, and an increase in the expression of NTPDase5 and mt-PCPH blocks this pathway and reduces the levels of apoptosis (Figure 2).

It was also observed that the overexpression of the NTPDase5 protein and more significantly the mt-PCPH actually decreases the intracellular concentration of ATP and confers resistance not only to stress-induced apoptosis but also to those induced by chemotherapy. The overexpression of these enzymes increased resistance of prostate tumor cells when in contact with cisplatin and of colorectal carcinoma cells when in contact with oxaliplatin [9, 22].

The proposed mechanism by which NTPDase5/mt-PCPH increases the neoplastic cell resistance to cisplatin is due to the ability of this protein to prevent the dephosphorylation of the kinase PKC*α* induced by chemotherapy. By keeping the PKC*α* phosphorylated at Thr638, this protein phosphorylates and stabilizes the antiapoptotic protein Bcl-2 at Ser^70^, making this enzyme resistant to cisplatin-induced proteasome degradation pathway, as suggested by Villar et al. [22]. Furthermore, it was observed that NTPDase5 interacts functionally with not only the PKC*α* but also the PKC*δ* protein, which was recognized as a key mediator in the NTPDase5 functions related to changes in the cell growth and invasive activity of the pancreatic tumor cells (Figure 2) [22].

Fang et al. [23] have demonstrated the involvement of the NTPDase5 also as an important link in the PI3K/PTEN loop-signaling pathway, which promotes cell growth and survival and is frequently found active in tumor cells. In this work, the authors found that PTEN knockout cells had increased expression of NTPDase5, and its overexpression is correlated with the activation of AKT, especially in cell lines of various tumor types and in primary tumor samples (Figure 2). These knockout cells also showed an increased degradation of ATP to AMP; however, as the substrates of NTPDase5 are UDP and ADP, it was found that the ATP degradation pathway occurs in the presence of the enzyme CMPK1, which removes a phosphate of ATP to phosphorylate an UMP molecule, generating UDP and ADP, respectively.

NTPDase5, by activating AKT, also plays a critical role in triggering the Warburg effect, leading to an increase in anaerobic glycolysis even in the presence of oxygen, increasing the levels of lactate and production of important macromolecules for cell proliferation, promoting angiogenesis and metastasis [23, 24]. Cells with activated PI3K/AKT pathway have a higher level of protein translation, which causes an overloading in the endoplasmic reticulum and increases the chances of a deficient folding process and consequent malformed proteins. The NTPDase5 is a protein present in the endoplasmic reticulum, and overexpression of this enzyme increases the degradation of UDP to UMP promoting protein N-glycosylation and folding, reducing the stress in the tumor cells endoplasmic reticulum [23, 24]. This mechanism allows growth factor receptors such as EGFR, IGFR, and HER-2 to be expressed and properly folded, maintaining high levels of these receptors in tumor cells. In fact,* NTPDase5* knockout in cells possessing active AKT impairs the expression and glycosylation of these receptors [23, 24].

In addition to participating in the PI3K/PTEN signaling pathway, which is overactive in approximately 90% of GBMs, NTPDase5 also plays an important role in the development of this cancer. This protein plays a modulatory role in the bioenergetics of this malignancy, increasing the catabolic efficiency of the aerobic glycolysis. In addition, when* NTPDase5* is suppressed, it causes a decrease in fatty acid oxidation and promotes an increase in the ATP influx and of the autophagic vacuoles in the cytoplasm [15].

Tumors of the respiratory system have a heterogeneity in what concerns the activation of Akt and PTEN inhibition. Those with this signaling pathway being active, however, are more resistant to treatments that involve starvation [25]. Indeed, suppression of the* NTPDase5* in lung carcinoma cells results in a decrease in Akt activity, decreased levels of IGF-IR growth factor receptor, and reduced cell proliferation under conditions of starvation, making these cells more susceptible to this type of treatment [25].

Finally, overexpression of* mt-PCPH* in colorectal carcinoma caused an increase in chemotherapy resistance [9]. The authors showed that a crucial event for this observation was a decrease in the intracellular levels of ATP [9]. However, no ATPase activity was observed in the mt-PCPH protein, probably due to a loss of conserved catalytic determinant regions in the truncated form, which may affect its tertiary structure and then the enzymatic function. This suggests that ATP degradation caused by the mutated protein may occur through interaction with other proteins and not through its NTPDase activity* per se*. Such findings can redirect all research involving the action of mt-PCPH in neoplastic processes and need to be further investigated [9].

## 4. Conclusions

In most of the types of cancers studied, the NTPDase5/mt-PCPH shows a change in its expression levels even in precursors of the malignant and benign lesions, which makes this protein a potential tool for early diagnosis of tumorigenesis. This enzyme has also been identified as a key element in a number of pathways known to be frequently activated in neoplastic processes and which give tumor cells a survival advantage when compared to healthy cells. In most cases, the participation of the NTPDase5/mt-PCPH occurs with a change in the intracellular ATP concentration and with participation of this enzyme in the phosphorylation and activation processes of proteins with antiapoptotic activity, conferring to the tumor cells resistance against apoptosis by stress or by chemotherapy treatments. The two main pathways related to the NTPDase5/mt-PCPH activity are the mTOR and the PI3K/PTEN signaling pathways, which are directly related, since the inhibition of PTEN results in a PI3K and consequent AKT overactivation, which in turn regulates the growth of tumor cells by different signaling pathways, one being its effect on mTOR (Figure 2).

This review included all the data published so far regarding the role of the proteins NTPDase5/mt-PCPH in cancer development and progression. Due to the scarcity of studies with NTPDase5/mt-PCPH, it is difficult to establish tissue- or cell-type-specific functions. However, it seems relatively well established that* ENTPD5* and mainly* mt-PCPH* expression are related with tumorigenic transformation. The findings presented in the studies reviewed raise the idea of using NTPDase5 as a possible target for cancer treatment.

## Figures and Tables

**Table 1:** NTPDase5 profile expression in tumor cell lines.

Author; year	Tumor cell line	Control group	Methods	NTPDase5 expression	mt-PCPH expression
Beckenkamp et al., 2014 [14]	Cervical cancer cell lines SiHa, HeLa, and C33A	Normal immortalized keratinocytes (HaCaT cells)	RT-PCR	Expressed in all cell lines with higher levels in SiHa cells than HeLa, C33A, and HaCaT cells	Not described
Zadran et al., 2012 [15]	Human brain tumor cell lines U87 and U87vIII	Not described	WB	Expressed in U87 and at higher levels in U87vIII	Not described
Villar et al., 2007 [16]	Human prostate tumor cells lines LNCaP, C42, and PC3	Nonneoplastic human prostatic epithelial cells (RWPE-1)	RT-PCR and WB	Not detected in RWPE-1 but it was highly detected in LNCaP and in both C42 and PC3 was expressed at lower levels	Not described
Regadera et al., 2006 [17]	Testicular germ cell tumors NCCIT (mutant Tp53) and NT2/D1 (wild-type Tp53)	Not described	WB	Both cell lines expressed several NTPDase5-immunorelated polypeptides (ranged 20–90 KDa)	Low molecular-size polypeptides were less abundant in NT2/D1 than NCCIT cells
Blánquez et al., 2004 [18]	Human benign and malignant neoplastic breast samples	Normal human breast tissue samples	WB	Both normal tissue and benign and malignant breast tumors samples showed the expression of the NTPDase5 protein	Only the more aggressive breast tumor samples expressed the mt-PCPH
Blánquez et al., 2002 [19]	Cell lines cultured from explants of laryngeal tumors (SCC) at stages II, III, and IV	Primary laryngeal epithelial cells (LECs) from the normal margin of surgical specimens	WB	Expression related directly to the evolution of the three grades of laryngeal dysplasia, characterized by increments of cell proliferation in parallel with changes in epithelial differentiation	LECs expressed more mt-PCPH than normal NTPDase5 and SCCs presented a loss in the mt-NTPDase5
Rouzaut et al., 2001 [12]	20 mammary tumor derived cell lines	Not described	WB	Detectable in 8 of the 20 cells lines	The mt-PCPH, after prolonged exposures, was detectable in all but two cell lines
Rouzaut et al., 2001 [12]	18 tumor cell lines derived from the central or peripheral nervous system	Not described	WB	Absent in 13 cell lines and barely detectable in 4 cell lines	Expressed in 13 cell lines
Rouzaut et al., 2001 [12]	6 colon tumor cell lines	Not described	WB	Expressed highly in all six cell lines	Expressed in 4 cell lines
Rouzaut et al., 2001 [12]	5 lung tumor cell lines	Not described	WB	Detectable at low levels in all five cell lines	Detectable at low levels in 4 cell lines
Rouzaut et al., 2001 [12]	1 pancreas tumor cell line	Not described	WB	Highly expressed	Detectable at low levels

Legend: U87vII: U87 glioblastoma cells transduced to express the epidermal growth factor receptor vIII; WB: Western blot.

**Table 2:** NTPDase5/mt-PCPH profile expression in clinical samples.

Author; year	Tissue study	Methods	Sampling size	Control group	NTPDase5 expression	mt-PCPH expression
Zadran et al., 2012 [15]	Primary glioblastoma multiforme (GBMs)	Tissue microarrays, IHC, and WB	140 patients	Adjacent normal brain	Elevated levels were observed in GBM cores when compared to adjacent normal tissues	Not described
Mikula et al., 2010 [20]	Adenocarcinomas and colonic adenomas	Mass spectrometry and qRT-PCR	5 adenocarcinomas; 12 colonic adenomas; 4 normal mucosas	Normal mucosa	Continuously downregulated in a progression from normal mucosa to adenocarcinoma	Not described
Villar et al., 2007 [16]	Prostate normal, hyperplasic, and tumor cells	IHC	63 patients	Normal human prostate	Not detected in normal prostate, detected slightly in HPB, and elevated in PIN and prostate carcinoma (samples with a *Gleason* score less *than 7* present lower levels)	Not described
Regadera et al., 2006 [17]	Testicular tumors	IHC	54 patients	Normal testicular tissue	Increased expression in testicular tumors relative to normal tissue; present in well-differentiated squamous epithelia and lost in dedifferentiated squamous cells	Not described
Blánquez et al., 2002 [19]	Laryngeal mild, moderate, and severe stages dysplastic lesions	IHC	59 patients	Normal laryngeal mucosa	Expressed at lower levels in severe than in mild dysplastic cases and at much lower levels than in the normal tissue	Not described
Blánquez et al., 2004 [18]	Human breast tumors	IHC and WB	54 patients	Normal human breast samples	Undetectable in normal and benign samples and increase in carcinoma *in situ* and more strongly in invasive ductal and lobular carcinoma	Absent in benign human breast and low molecular weight polypeptides in ductal and lobular carcinoma
Solanas et al., 2002 [13]	Rat mammary benign and malignant tumors induced	IHC and WB	35 malignant tumors; 19 benign tumors	Normal rat mammary gland	Tumor samples showed higher levels and were more expressed in the malignant tumors	Tumor samples showed decrease in the mt-NTPDase5 expression when compared with the normal tissue

Legend: HPB: hyperplasia prostate benign; PIN: prostatic intraepithelial neoplasia; IHC: immunohistochemistry; WB: Western blot.
